# Woman aspects of behavior addiction, including gambling

**DOI:** 10.1192/j.eurpsy.2023.133

**Published:** 2023-07-19

**Authors:** Z. Kalo

**Affiliations:** Institute of Psychology, ELTE University, Budapest, Hungary

## Abstract

**Abstract:**

Behavioral addictions, also known as non-substance or non-drug addictions, refer to a range of compulsive behaviors that individuals engage in despite the negative consequences that result from these behaviors. Research on behavioral addiction in women has shown that women are at a higher risk for certain types of behavioral addictions, such as shopping addiction and internet addiction, compared to men. Studies suggest that women may be more susceptible to these types of addictions because of social and cultural factors, such as societal expectations of women to be nurturing and caregiving, which may lead them to use these behaviors as a form of coping mechanism. There is also evidence that women are more likely to experience shame and guilt as a result of their addiction, which can make it more difficult for them to seek help and support. Gambling among women has traditionally been less common than among men however, this trend is changing. Survey have shown that the number of women who gamble is on the rise, and that they are becoming increasingly diverse in terms of age, income, social background. Research studies have found that the rate of problem gambling among women is lower than men, but that women tend to develop gambling problems more quickly than men. This is thought to be due to a number of factors, including women’s greater vulnerability to stress and depression, as well as the fact that women are more likely to have a history of trauma or abuse. Overall, research on behavioral addictions among women is still a relatively new field, and there is a need for more studies to be conducted in order to better understand the unique factors that contribute to the development of these types of addictions in women.

**Disclosure of Interest:**

None Declared

